# Giant omphalocele associated pulmonary hypertension: A retrospective study

**DOI:** 10.3389/fped.2022.940289

**Published:** 2022-09-09

**Authors:** Tai-Xiang Liu, Li-Zhong Du, Xiao-Lu Ma, Zheng Chen, Li-Ping Shi

**Affiliations:** Department of NICU, Zhejiang University School of Medicine, National Clinical Research Center for Child Health, National Children’s Regional Medical Center, Children’s Hospital, Hangzhou, China

**Keywords:** giant omphalocele, omphalocele, pulmonary hypertension, neonates, infants

## Abstract

**Background:**

Omphalocele is a common congenital defect of the abdominal wall, management of giant omphalocele (GO) is particularly for pediatric surgeons and neonatologists worldwide. The current study aimed to review and summarize the clinical features and prognosis in neonates with GO complicated with pulmonary hypertension (PH), which is associated with increased mortality, while in hospital.

**Materials and methods:**

Medical records of infants with GO between July 2015 and June 2020 were retrospectively analyzed. The patients enrolled were divided into PH and non-PH groups based on the presence or absence of PH, and patients with PH were divided into death and survival groups based on survival status. Clinical characteristics and outcomes were compared between groups, respectively. The risk factors for PH were analyzed by binary logistic regression.

**Results:**

In total, 67 neonates were identified as having GO and 24 (35.8%) were complicated with PH. Infants with PH were associated with intubation within 24 h after birth (*p* = 0.038), pulmonary dysplasia (*p* = 0.020), presence of patent ductus arteriosus (PDA; *p* = 0.028), a staged operation (*p* = 0.002), longer mechanical ventilation days (*p* < 0.001), oxygen requirement days (*p* < 0.001), parenteral nutrition (PN) days (*p* < 0.001), length of neonatal intensive care unit (NICU) or hospital stay (*p* = 0.001 and 0.002, respectively), and mortality (*p* = 0.001). The results of multivariable logistic regression analysis revealed that a staged operation was independently associated with PH. In addition, PH patients with lower birth weight, higher peak of pulmonary arterial systolic pressure, and refractory to pulmonary vasodilators (PVD) had increased mortality.

**Conclusion:**

Pulmonary hypertension is a serious complication and significantly increases the mortality and morbidities in infants with a GO. In addition, early and serial assessment of PH by echocardiography should be a routine screening scheme, especially in the neonatal omphalocele population who required a staged surgical repair. Clinicians should be aware that infants with PH who had low weight, severe and refractory PH have a higher risk of death.

## Introduction

An omphalocele is a congenital abdominal wall defect that usually results in herniation of the abdominal organs, such as the liver, spleen, stomach, and intestine within a sac, and the condition has an estimated incidence of 1–3.8 per 10,000 life births (1, 2). Omphalocele can be generally classified into three types, i.e., small, giant, and ruptured, according to the size of the abdominal wall defect and/or its contents (3). Previous studies indicated that the size of an omphalocele, the increased viscero-abdominal disproportion, and the associated chromosomal or other important organ abnormalities are the main causes of death (4–6). Compared with a small omphalocele, infants with a giant omphalocele (GO) have a higher mortality rate of 20–25% (7, 8).

In recent years, clinicians have found that GO is associated with abnormalities in the pulmonary parenchyma and vasculature that lead to pulmonary hypoplasia and pulmonary hypertension (PH) (9–12). Moreover, PH is a severe and potentially fatal complication causing respiratory insufficiency, which requires aggressive ventilator support during the early postnatal period (13, 14). Based on these clinical questions, we carried out a retrospective analysis for characterizing the clinical features and prognosis of infants with a GO-associated PH.

## Patients and methods

**FIGURE 1:**
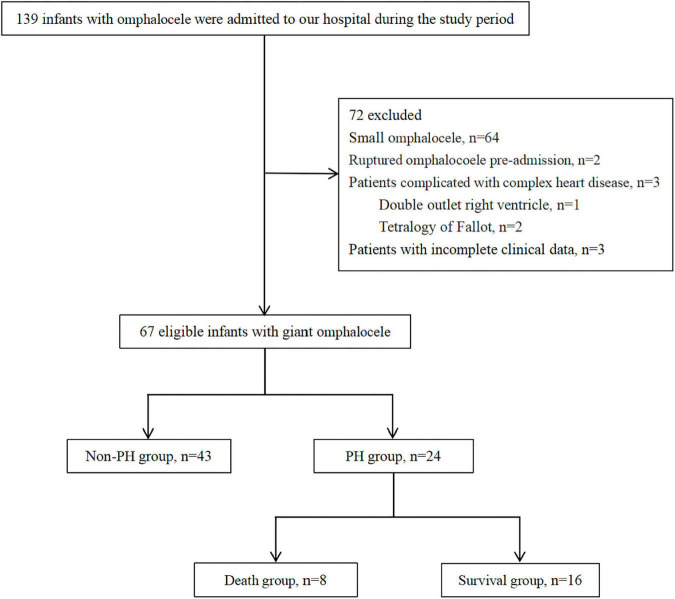
Flowchart of participants.

With the approval of the Institutional Review Board (IRB No. 2021-IRB-012), a retrospective study was performed on infants with congenital GO admitted to the neonatal intensive care unit (NICU) of the Children’s Hospital, Zhejiang University School of Medicine between July 2015 and June 2020. The inclusion criteria for the study were all patients with a diagnosis of GO during the study period. Exclusion criteria were as follows: (1) small omphalocele; (2) ruptured omphalocele pre-admission; (3) congenital heart disease except for atrial septal defect (ASD), patent foramen ovale (PFO), patent ductus arteriosus (PDA), or small ventricular septal defect (VSD); and (4) patients with incomplete clinical data. The patients enrolled in the study were divided into PH and non-PH groups and patients with PH into death and survival groups according to the survival status (Figure 1). The patients’ demographics, delivery information, cyst contents, associated abnormalities, neonatal care, surgical repair, respiratory and nutritional support requirements, and short-term prognosis of PH were recorded.

### Definitions and interventions

We routinely used echocardiography to evaluate cardiac structure and pulmonary artery systolic pressure (PASP) in neonates with GO during the first week after birth and repeated once weekly in patients with clinical or echocardiographic evidence of PH or more frequently if clinically indicated, such as worsening of shunting, increased ventilator requirements, or other signs of clinical deterioration. PASP was estimated by measuring tricuspid regurgitation velocity (TRV) or calculated from the systemic pressure and the shunt pressure difference in the presence of intra-cardiac or extracardiac shunt. When there is no right ventricle outflow tract obstruction, PASP is equal to the right ventricular systolic pressure (PASP = 4 × (TRV_max_)^2^ + right atrium pressure) (15). Generally, TRV < 2.5 m/s is considered normal (16). As the gold standard, right cardiac catheterization (RHC) should be performed when PH is clinically considered and cannot be accurately measured by echocardiography. PH was defined on echocardiography as the presence of tricuspid regurgitant jet with an estimated systolic pulmonary artery pressure (PAP) > 2/3 systemic systolic blood pressure and/or right-to-left or bidirectional flow at ductal and/or atrial level (17, 18). Once PH was diagnosed, pulmonary vasodilators (PVDs) were administered individually that included inhaled nitric oxide (iNO), treprostinil, milrinone, sildenafil, or bosentan.

The therapeutic effect of PVD on PH was echocardiographic improvement in PH. Echocardiographic improvement was defined as a 20% decrease in the absolute tricuspid regurgitation gradient or transform right-to-left or bidirectional flow into the left-to-right flow at ductal and/or atrial level between serial echocardiograms (19). Ineffectiveness was defined as without the above echocardiographic improvement or death occurred rapidly due to PH after treatment. Echocardiographic data were recorded, when possible, during the course of PVD treatment: time of introduction, 24–48 h after treatment, every other week after treatment and the disease worsen, or at the cessation of treatment or before discharge.

Giant omphalocele was defined as a large abdominal evisceration with a covering membrane containing more than 50–75% of the liver or an abdominal wall defect with a size of more than 5 cm (8). Pulmonary hypoplasia was characterized by a long, slight bell-shaped, and narrow chest with a marked reduction in lung volume and typical radiographic signs (20).

Patent ductus arteriosus was defined as the existence of persistent ductus arteriosus as demonstrated by echocardiography performed beyond 72 h of life in near-term and term infants (21). Respiratory system abnormalities included pulmonary hypoplasia, airway (tracheal bronchus and vascular loop), and diaphragm (diaphragmatic hernia and eventration). Digestive system abnormalities included intestinal malrotation, Meckel’s diverticulum, and mesenteric hiatal hernia. Urogenital system abnormalities included indirect inguinal hernia and cryptorchidism.

In addition, we defined the liver and spleen as solid organs and the stomach, gallbladder, and intestine as hollow organs. Abdominal wall closure was performed by either disposable closure or staged repair that depends on the development of the abdominal cavity and the size of the omphalocele.

## Statistical analysis

Statistical analysis was performed using the IBM SPSS version 21 (IBM SPSS Inc., Chicago, United States). The Student’s *t*-test and the Mann–Whitney *U* tests were used for continuous variables according to whether the data conformed to normal or non-normal distributions, respectively. *X*^2^ test or Fisher exact test was used for categorical variables as appropriate. Data were presented as mean value ± standard deviation (SD), median with quartile range [M (Q1, Q3)], or percentages. All probability (*p*) values were two-tailed and a value < 0.05 was considered statistically significant.

In addition, we used the occurrence of PH as the dependent variable and variables with a statistically significant difference between the two groups (i.e., intubation within 24 h after birth, pulmonary hypoplasia, PDA, or a staged operation) and variables showing no statistical difference but reported in the previous literature to be related to PH (i.e., small for gestational age (SGA), perinatal asphyxia, or a solid/hollow organ in the sac) as the independent variables. The binary logistic regression analysis was performed using the forward logistic regression method, and the results are expressed as odds ratio (OR) with 95% CI.

The sample size was calculated by online software^1^ and based on a retrospective literature review that concluded that in 48% of infants with omphalocele, the condition is complicated by PH. We hypothesized that 30% of infants had suffered from PH during hospitalization, considering the different definitions of PH, with 80% power, α = 0.05, and the 2-tailed test. The estimated total number of patients was 58, and the actual power was calculated to be more than 80%.

## Results

A total of 67 infants with GO met the inclusion criteria and were identified in the retrospective analysis. In total, 35.8% (24/67) of infants presented echocardiographic evidence meeting the criteria for a diagnosis of PH, while the remaining 64.2% (43/67) did not have evidence of PH.

Compared with the non-PH group, the PH group had a higher proportion of intubation within 24 h after birth (*p* = 0.038), pulmonary hypoplasia (*p* = 0.020), a PDA (*p* = 0.028), and a staged operation (*p* = 0.002). The other clinical characteristics were not significantly different between the two groups (*p* > 0.05; Table 1). The results of binary regression analysis showed that the staged operation was independently associated with PH in infants with a GO (*p* = 0.010; Table 2).

**TABLE 1:** Comparison of the baseline characteristics of the study cohort by the presence of PH.

Variables	PH group (*n* = 24)	Non-PH group (*n* = 43)	*P-*value
Male gender	16 (66.7%)	20 (46.5%)	0.113
Gestational age (wk)	38 (36.3–38.3)	38 (36.1–38.7)	0.942
Birth weight (g)	2834 ± 690	2693 ± 581	0.378
SGA	2 (8.3%)	7 (16.3%)	0.589
Perinatal asphyxia	8 (33.3%)	6 (14%)	0.061
Intubation within 24 h after birth	6 (25%)	2 (4.7%)	0.038*
Solid organ in the sac	24 (100%)	43 (100%)	NA
Hollow organ in the sac	19 (79.2%)	27 (62.8%)	0.166
Respiratory system abnormalities	21 (87.5%)	27 (62.8%)	0.031*
Pulmonary hypoplasia	21 (87.5%)	26 (60.5%)	0.020*
Tracheal bronchus	2 (8.3%)	0 (0%)	0.125
Vascular loop	2 (8.3%)	0 (0%)	0.125
Diaphragmatic hernia and eventration	2 (8.3%)	1 (2.3%)	0.290
Digestive system abnormalities	20 (83.3%)	32 (74.4%)	0.401
Intestinal malrotation	20 (83.3%)	29 (67.4%)	0.159
Meckel’s diverticulum	3 (12.5%)	4 (9.3%)	0.695
Gallbladder agenesis	2 (8.3%)	3 (7%)	>0.999
Mesenteric hiatal hernia	0 (0%)	1 (2.3%)	>0.999
Urogenital system abnormalities	6 (25%)	4 (9.3%)	0.149
Indirect inguinal hernia	5 (20.8%)	3 (7%)	0.124
Cryptorchidism	2 (8.3%)	2 (4.7%)	0.614
Beckwith-wiedemann syndrome	1 (4.2%)	1 (2.3%)	>0.999
PDA	7 (29.2)	3 (7%)	0.028*
Age at surgery (d)	2 (2–5)	2 (1–3)	0.144
Staged operation	7 (29.2)	1 (2.3)	0.002

IQR, interquartile range; PDA, patent ductus arteriosus; PH, pulmonary hypertension; SD, standard deviation; SGA, small for gestational age; NA, not applicable. *Represents statistically significant, *p* < 0.05.

**TABLE 2:** Logistic regression analysis of risk factors of GO-associated PH.

Variable	β	*P-*value	OR	95% CI
Staged operation*	2.850	0.010	17.294	1.975–151.424
Constant	–0.904	0.002	0.405	

CI, confidence interval; GO, giant omphalocele; OR, odds ratio; PH, pulmonary hypertension. *Represents statistically significant, *p* < 0.05.

In terms of clinical outcome indicators, PH was found to be significantly associated with the duration of mechanical ventilation (*p* < 0.001), oxygen requirement days (*p* < 0.001), length of NICU stay (*p* = 0.001), and length of hospital stay (*p* = 0.002). In addition, the requirement for supplemental oxygen at home in the PH group was higher than that in the non-PH group (20.8 vs. 7.0%), although this difference was not statistically significant (*p* > 0.05). In addition, digestive system abnormalities were diagnosed in the majority of patients in both patients’ cohorts, with a requirement for parenteral nutrition (PN) support lasting for 31 d in the PH group and 14 days in the non-PH group, respectively (*p* < 0.001). It should be noted that mortality was also increased significantly in the PH group (*p* = 0.001; Table 3).

**TABLE 3:** Comparison of clinical outcomes of the study cohort by the presence of PH.

Variables	PH group (*n* = 24)	Non-PH group (*n* = 43)	*P-*value
Duration of mechanical ventilation (days)	11 (5–42)	3 (1–6)	<0.001*
Oxygen requirement days (days)	39 (21–62)	15 (6–31)	<0.001*
Requirement for supplemental oxygen at home	5 (20.8%)	3 (7%)	0.065
PN days (days)	31 (22–48)	14 (10–19)	<0.001*
Nasal feeding home	1 (4.2%)	1 (2.3%)	>0.999
Length of NICU stay (days)	28 (14–51)	11 (8–20)	0.001*
Length of hospital stay (days)	40 (24–65)	19 (15–29)	0.002*
Death	8 (33.3%)	1 (2.3%)	0.001*

IQR, interquartile range; NICU, neonatal intensive care unit; PH, pulmonary hypertension; PN, parenteral nutrition. *Represents statistically significant, *p* < 0.05.

Table 4 provides a description of the clinical characteristics of infants with PH in the death and survival groups. Patients in the death group had a lower birth weight (*p* = 0.019) and a higher systolic PAP (*p* = 0.003). In terms of treatment, 1 patient suffered from sudden death due to PH crisis without PVD therapy, while 19 infants received two or more types of PVD and 4 underwent PDA ligation. As for the efficacy of the drug, the patients in the death group had a worse response to the PVD therapy (*p* = 0.004).

**TABLE 4:** Clinical characteristics of death and survival groups in infants with PH.

	Death group (*n* = 8)	Survival group (*n* = 16)	*P-*value
Gestational age (wk)	37 (32.7–38.1)	38 (37.5–38.6)	0.114
Birth weight (g)	2379 ± 787	3061 ± 525	0.019*
Intubation within 24 h after birth	3 (37.5%)	3 (18.8%)	0.362
Time of diagnosis of PH (days)	8 (1–78)	5 (3–7)	0.516
PH diagnosed within 7 days after birth	4 (50%)	12 (75%)	0.363
PASP (mmHg)	86.4 ± 21.7	61.3 ± 15.4	0.003*
Systolic SBP (mmHg)	76.5 ± 9.9	71.3 ± 7.1	0.152
**Types of PVD**			
Single type of PVD	0 (0%)	4 (25%)	0.273
Two types of PVD	3 (42.9%)	7 (43.8%)	>0.999
Three or more types of PVD	4 (57.1%)	5 (31.3%)	0.363
**Therapeutic effect of PH**			
Improvement	3 (42.9%)	16 (100%)	0.004*
Ineffectiveness	4 (57.1%)	0 (0%)	0.004*
Duration of PVD (days)	21 ± 36	38 ± 28	0.257
PDA	2 (25%)	5 (31.3%)	>0.999
PDA ligation	0 (0%)	4 (25%)	0.262

IQR, interquartile range; PASP, pulmonary arterial systolic pressure; SBP, systemic blood pressure; PDA, patent ductus arteriosus; PH, pulmonary hypertension; PVD, pulmonary vasodilators; SD, standard deviation. *Represents statistically significant, *p* < 0.05.

## Discussion

This study reviewed and summarized the clinical data of infants with GO complicated with PH who were admitted to a national regional medical center in China. We found that infants with PH have a higher proportion of intubation within 24 h after birth, pulmonary hypoplasia, presence of a PDA, a staged operation for an omphalocele and were associated with the requirement for respiratory support, prolonged hospital stay, and increased mortality. The results of regression analysis showed that a staged operation was an independent risk factor for PH. In addition, infants with lower birth weight, higher degree of systolic PAP, and refractory to PVD therapy were more likely to increase mortality.

Giant omphalocele-associated PH has been reported to be a severe, even potentially life-threatening clinical complication with high morbidity and mortality in previous studies (22), similar to the results of our study. However, the pathogenesis of PH in patients with GO remains unclear. At present, it is considered that it may be related to pulmonary dysplasia, pulmonary vascular smooth muscle remodeling, and abnormal vascular tension (11). This was confirmed by a case of omphalocele complicated with severe PH, with the final pathological diagnosis consistent with congenital alveolar capillary dysplasia and characterized by a fatal developmental lung disorder observed in neonates and infants (9). In addition, prenatal ultrasound and magnetic resonance imaging (MRI) had been applied to assess the total lung volume and pulmonary hypoplasia in infants with an omphalocele, suggesting that the lung volume and gas exchange area were significantly reduced (23–25). Animal experiments further indicated that an abdominal wall defect does in fact affect the differentiation between type I alveolar epithelial cells and type II alveolar epithelial cells (26). Our study found that the pulmonary dysplasia diagnosed by typical radiographic signs was far more common in infants with PH who suffered from a higher proportion of the requirement for ventilator and oxygen support, which suggested that GO-associated PH is indeed a certain degree of primary pulmonary developmental abnormalities.

Unlike many other diseases, an optimal surgical repair approach to the omphalocele remains controversial and frequently requires multi-disciplinary consideration of various factors, such as the size of abdominal wall defect, presence of herniated solid organs, presence of associated abnormalities, and the severity of associated pulmonary hypoplasia and PH (27, 28). Early primary closure of the defect may lead to a sudden increase in intra-abdominal pressure and abdominal compartment syndrome, which presents as acute kidney injury, respiratory, and hemodynamic deterioration in those GO patients with a small abdominal cavity (29, 30). In our study, infants with a GO required staged surgical closure of the defect, which was significantly correlated with PH, possibly due to the potential small abdominal cavity and a high viscero-abdominal disproportion, which may modify diaphragm function and mobility, causing secondary pulmonary dysplasia and respiratory compromise (10).

Pulmonary hypertension was considered to be independently associated with mortality in patients with omphalocele (11–13). Our study further confirmed that patients with PH who had lower birth weight, higher PASP, and refractory to PVD therapy were having increased mortality. As for treatment, combined use of PVD with different sites of action should be considered as a consequence of the multiple mechanisms involved in the pathogenesis of PH (31, 32). We preferred to administer iNO or remodulin when infants were in a critical condition, followed by oral sildenafil and/or bosentan for further treatment. Significantly, PDA is a common cardiovascular defect characterized by a large volume of transductal left-to-right shunt that may increase pulmonary blood flow and the left-side heart volume loading, causing a further increase in PAP and cardiac insufficiency (21). In our study, the symptoms that presented as persistent ventilator or oxygen dependence in four patients diagnosed with PH accompanied by symptomatic PDA were partially alleviated after transthoracic PDA ligation, despite a lack of consensus regarding the indication for PDA surgical intervention in this population (33).

## Limitations

First, the sample capacity of the study was hampered by excluding small-sized omphalocele. Second, we only diagnosed pulmonary dysplasia through typical radiological signs rather than lung biopsy to determine the development of pulmonary parenchyma and vasculatures. Third, the present study lacks follow-up on the long-term prognosis that includes pulmonary and neurological function in patients. Finally, the study lacks innovation to a certain extent, because the genetic and molecular biological analyses were not performed in participants.

## Conclusion

In conclusion, PH is a serious complication and significantly increases the mortality and morbidities in infants with a GO. Early and serial assessment of PH by echocardiography should be a routine screening scheme, especially in the neonatal omphalocele population who required a staged surgical repair. Furthermore, clinicians should be aware that infants with PH who had low weight, severe and refractory PH, have a higher risk of death.

## Data availability statement

The original contributions presented in this study are included in the article/supplementary material, further inquiries can be directed to the corresponding author.

## Ethics statement

The studies involving human participants were reviewed and approved by Institutional Review Board of Medical Ethics of the Children’s Hospital, Zhejiang University School of Medicine. Written informed consent from the participants’ legal guardian/next of kin was not required to participate in this study in accordance with the national legislation and the institutional requirements.

## Author contributions

T-XL and L-PS were designed by the ideas and protocol of this study. T-XL, ZC, and X-LM were obtained by medical records. T-XL completed the manuscript and worked for the statistical analysis. L-PS and L-ZD revised the article. All authors contributed to the article and approved the submitted version.

## Footnotes

1
https://www.biostats.cn/samplesize/
